# Prescription Patterns of Analgesic Drugs in the Management of Pain Among Palliative Care Patients at a Tertiary Hospital in Oman: A Retrospective Observational Study

**DOI:** 10.7759/cureus.41501

**Published:** 2023-07-07

**Authors:** Abdullah Al Lawati, Nasser Al Wahaibi, Yousuf Al Suleimani

**Affiliations:** 1 College of Medicine and Health Sciences, Sultan Qaboos University, Muscat, OMN; 2 Pharmacology & Clinical Pharmacy, Sultan Qaboos University, Muscat, OMN

**Keywords:** painkillers, pain management, prescription pattern, analgesic treatment, palliative care

## Abstract

Objectives

Analgesic drugs are commonly used to alleviate the pain experienced by palliative care (PC) patients. Thus, we sought to determine the prescription patterns of analgesic drugs in the management of pain among haematology and oncology palliative care patients at Sultan Qaboos University Hospital (SQUH) and then see if they were following the World Health Organization (WHO) guidelines.

Methods

A retrospective observational cross-sectional study was conducted, and adult PC patients prescribed analgesics for pain relief between January 2018 and January 2021 at SQUH constituted the sample. Data were collected from patients' electronic medical records using the SQUH TrakCare system. The data was then presented descriptively using graphs and tables.

Results

Data from 200 PC patients were analyzed. Breast cancer was the most common malignancy, with 73 (36.5%) patients diagnosed with it. Severe pain was the most reported degree of pain, with exactly 100 (50.0%) patients experiencing it. More patients experienced mild pain than moderate pain. Opioids were the most prescribed analgesics, followed by analgesics and antipyretics, anticonvulsants, and finally non-steroidal anti-inflammatory drugs (NSAIDs). Paracetamol was the most prescribed analgesic for pain overall, with 127 (63.5%) patients utilizing it. For severe pain, morphine was the most prescribed analgesic, with 65.0% of patients using it. Fentanyl and pregabalin, the strongest two analgesics, increased in prescription for severe pain compared to mild and moderate pain, with both being prescribed to 23.0% of patients suffering from severe pain. The oral route of administration was the most prescribed, with 128 (64.0%) utilizing it.

Conclusion

This study showed the prescription patterns of analgesic drugs for palliative care patients at SQUH. The findings were similar to those of other studies, though there were some differences. The prescription patterns of analgesic drugs prescribed for the various pain levels among PC patients were found to be in accordance with the WHO guidelines.

## Introduction

Palliative care (PC) is a treatment approach aimed at improving the quality of life of patients with life-threatening illnesses through the prevention and relief of their pain [1]. Palliative care has recently become an essential component of the healthcare systems of many countries, as it has many benefits both to the patient and the healthcare system through reducing healthcare costs [2]. However, a report published by the World Health Organization (WHO) stated that only about 14% of patients who require palliative care receive it, and thus there needs to be more done by the world and the region concerning integrating palliative care in the healthcare system [1].

Many diseases are associated with palliative care patients, including advanced cancer, chronic obstructive pulmonary diseases (COPD), congestive heart failure (CHF), and end-stage renal diseases, with cancer specifically being the most common one [3]. Palliative care patients experience various symptoms, including pain, confusion, vomiting, anxiety, breathlessness, and depression [4]. Pain is the most common symptom experienced by palliative care patients [5], and one systematic review concludes that a median of 41% of CHF patients and 68% of COPD patients with palliative conditions have significant manifestations of it [6]. Pain can be physical or psychological and is one of the main determinants of a patient's quality of life and well-being. Many researchers consider it to be the "fifth vital sign" [7]. Thus, given the high prevalence of pain among PC patients, it is evident that a management approach is needed to relieve the pain of PC patients and improve their quality of life (QoL), which is where analgesic drugs become useful.

Analgesic drugs, commonly referred to as painkillers, work to alleviate a patient's pain. There are many ways to classify analgesic drugs; one classification is based on the receptor they act on. These include opioids and non-opioids. Opioids include codeine, morphine, fentanyl, and methadone; non-opioids include paracetamol and nonsteroidal anti-inflammatory drugs (NSAIDs) [8]. Another classification for analgesics, and one that is commonly used in practice, is the WHO analgesics ladder. This ladder classifies analgesics based on their strength; it is a three-step guideline that includes the progression from weaker to stronger analgesics based on the severity of pain experienced by the patient [9]. Weaker drugs such as paracetamol are the first-line drugs and thus found at the bottom of the ladder, whereas stronger drugs such as morphine are placed at the top of the ladder [10]. Quite recently, a modified WHO analgesic ladder has been proposed, placing anticonvulsants such as pregabalin at the top [11]. This is of significance, especially given that some patients do not respond well to morphine, the strongest drug in the original WHO ladder, with a study concluding that 30% of cancer patients did not respond well to morphine due to its intolerable side effects and inadequate pain relief [12]. Analgesic drugs are the most commonly prescribed medications for palliative care [13]. They can be used either as monotherapy or in combinations of two, three, and sometimes four-drug combinations [14], demonstrating the many options available for palliative therapy.

This study aimed to identify the prescription patterns of analgesic drugs among oncology and hematology palliative care patients at Sultan Qaboos University Hospital (SQUH) and then compare those findings with those of other hospitals worldwide. The rationale for conducting this project is that there is limited data currently on the analgesic drug prescription patterns in Oman, so this study would fill the gap through the experience of a single center (SQUH). In addition, the findings of this study give healthcare providers in Oman a clear picture of the current prescription practices and allow them to determine if there needs to be any modification or improvement to them. Lastly, the findings of this project will also provide a platform and basis for comparison with other hospitals and centers worldwide. Ultimately, this could all lead to improvements in the quality of healthcare in Oman and improvements in the patient's quality of life.

## Materials and methods

This study is a retrospective observational cross-sectional study conducted at Sultan Qaboos University Hospital, Muscat, Oman. Adult patients (≥ 18 years) with palliative conditions who were prescribed analgesics between January 2018 and January 2021 were eligible for this study. In addition, only PC patients whose diseases were of oncological or hematological origin were considered, as many hospitals worldwide group oncology and hematology patients under one unit rather than separating them, so this would allow for a more accurate comparison. Patients initially admitted for PC who no longer needed it were excluded from this study. Data was extracted through the patients' medical records using the SQUH TrakCare system and included the following: demographic data, medical history, diagnosis, severity of pain as reported through the clinical notes, outcome, and details of prescribed analgesics.

Using the sample size for the population mean formula at a confidence level of 95% and a margin of error of 5%, a total sample size of 195 patients was required for this study; however, data from 200 patients was collected. The collected data was analyzed using IBM SPSS Statistics for Windows, Version 27.0 (Released 2020; IBM Corp., Armonk, New York, United States). The data was presented as the mean with the standard deviation (SD), percentages, and range. Variables such as drug classes, routes of administration, opioid combinations, and many others were considered categorical data. Ethical approval was obtained from the Medical Research and Ethics Committee (MREC #2508) at the College of Medicine and Health Sciences, Sultan Qaboos University.

## Results

Data from 200 patients was collected, of whom 81 (40.5%) were males and 119 (59.5%) were females. The mean age of the patients was 59.00 ± 13.60 years, with the youngest patient being 24 years of age and the oldest one being 93. Patients with an oncological malignancy made up most of the sample, representing 95.50%. In contrast, patients with hematological malignancies made up the remaining 4.50% of the sample. None of the patients in the sample had both malignancies. Severe pain was the most reported regarding pain levels, with exactly 100 (50.0%) patients experiencing it. More patients experienced mild pain than moderate pain, with 58 (29.0%) patients experiencing mild pain and 42 (21.0%) patients experiencing moderate pain, respectively. The outcome of death was higher than discharge, with 104 (52.0%) patients passing away. Hypertension was the most commonly reported comorbidity, with 34.5% of the sample hypertensive. The demographic data of the sample is displayed in Table 1.

**Table 1:** Patient demographics Demographic data of the entire sample is represented as a mean and percentage of the sample across multiple factors. SD: standard deviation Total number: 200

Demographic		Mean ± SD (range) or no. %
Age	Years	59.00 ± 13.60 (24-93)
Sex	Males	81 (40.5%)
	Females	119 (59.5%)
Pain	Mild	58 (29.0%)
	Moderate	42 (21.0%)
	Severe	100 (50.0%)
Outcome	Discharge	96 (48.0%)
	Death	104 (52.0%)
Comorbidities	Hypertension	69 (34.5%)
	Diabetes	51 (25.5%)
Risk factors	Smoking	23 (11.5%)
Malignancy type	Oncological	191 (95.5%)
	Hematological	9 (4.50%)

**Figure 1:**
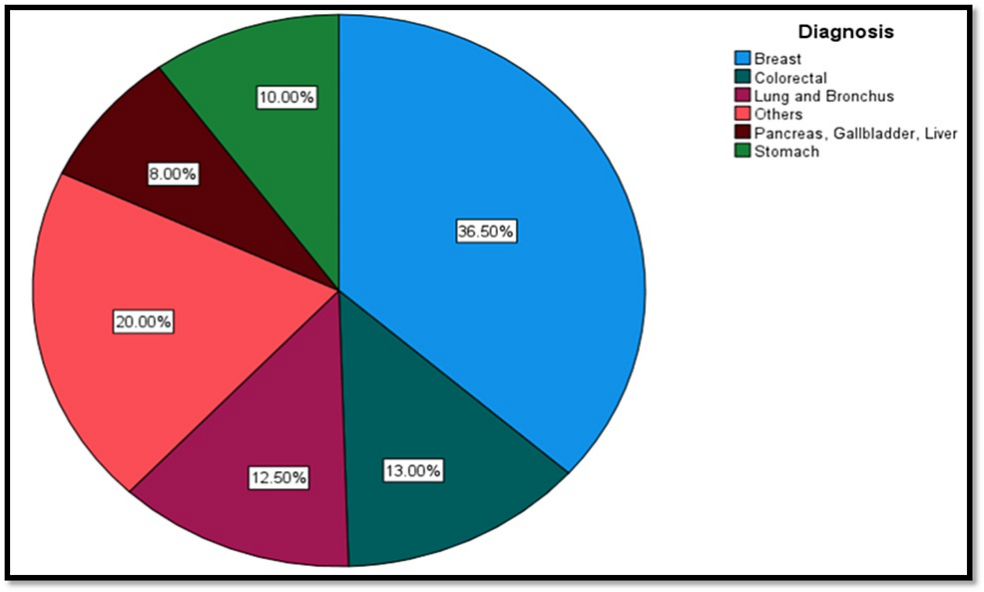
Most common diseases of the sample

As shown in Figure 1, breast cancer was the most common type of malignancy in the sample, with 73 (36.5%) diagnosed. Colorectal cancer was the second most common, with 26 (13.0%) patients suffering from it. This was followed by respiratory (lung and bronchus) malignancies, diagnosed in 25 patients. Forty (20.0%) patients were classified as 'others' in Figure 1, representing the culmination of various types of cancer.

As per Table 2, paracetamol was the most prescribed analgesic, with 127 (63.5%) patients utilizing it. In terms of analgesic drug classes, opioids were prescribed the most, as they were prescribed 169 times. This was followed by analgesics and antipyretics (127), then anticonvulsants (25), and lastly, NSAIDs (17). Within the class of NSAIDs, aspirin was the most prescribed, followed by diclofenac, ibuprofen, and celecoxib, which were prescribed equally. Morphine (±sulfate) was the most utilized opioid analgesic, with 73 patients using it. Tramadol, utilized by 61 patients, followed this; fentanyl, utilized by 26 patients; and codeine, utilized by nine patients. Despite the availability of multiple anticonvulsant analgesic drugs, pregabalin was the only drug prescribed to the sample, where 25 (12.5%) palliative care patients were given it.

**Table 2:** List of all analgesic drugs utilized by PC patients in the sample Classes of analgesics, including specific drugs within each class, that were utilized by the sample represented as number and percentage of patients utilizing the drugs. NSAIDs: non-steroidal anti-inflammatory drugs

Group of drugs (class)	Name of drug	Number and percentage of patients utilizing the drug
Analgesics & antipyretics	Paracetamol	127 (63.5%)
NSAIDs	Aspirin	13 (6.50%)
	Diclofenac	2 (1.00%)
	Ibuprofen	1 (0.50%)
	Celecoxib	1 (0.50%)
Opioids	Morphine (±Sulfate)	73 (36.5%)
	Tramadol	61 (30.5%)
	Fentanyl	26 (13.0%)
	Codeine	9 (4.50%)
Anticonvulsants	Pregabalin	25 (12.5%)

**Figure 2:**
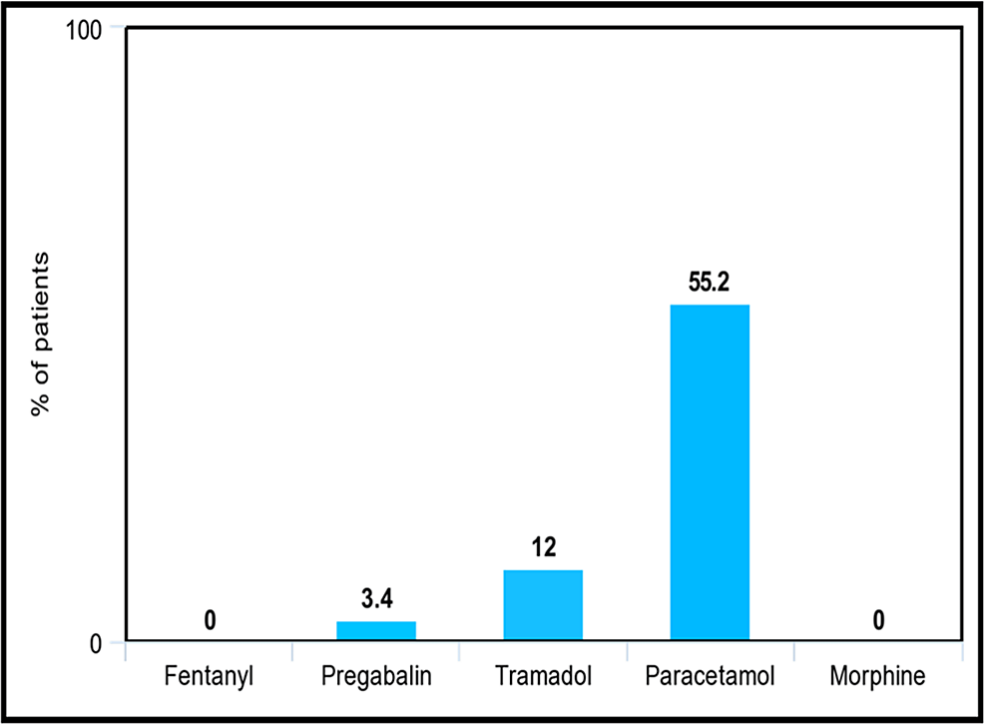
Most common analgesics prescribed for mild pain as a percentage of patients The specific analgesic drugs that were utilized by patients experiencing mild pain, represented by the percentage of patients utilizing them as shown in this bar chart.

Figure 2 represents the most common analgesics prescribed for mild pain. Paracetamol was the most prescribed drug and was utilized by 55.2% of patients suffering from mild pain. Stronger opioids were generally not prescribed, except for pregabalin, which was prescribed for 3.4% of the patients.

**Figure 3:**
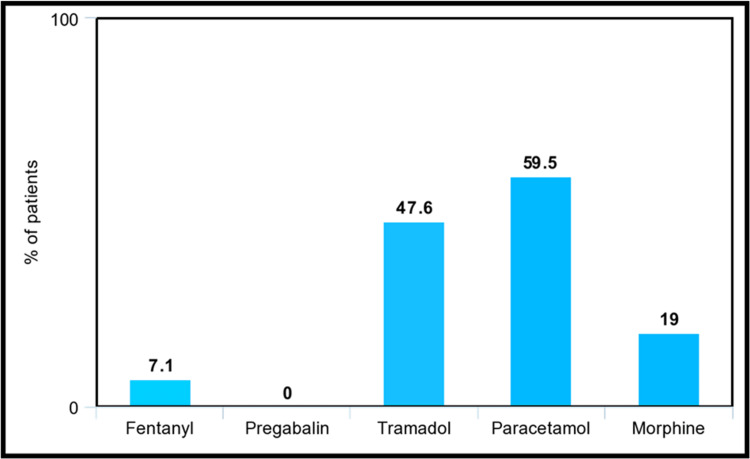
Most common analgesics prescribed for moderate pain as a percentage of patients The specific analgesic drugs that were utilized by patients experiencing moderate pain, represented by the percentage of patients utilizing them as shown in this bar chart.

In Figure 3, for moderate pain, paracetamol was once again the most prescribed drug, with 59.5% of the patients using it. However, tramadol prescriptions increased, with 47.6% of patients utilizing it. In addition, stronger opioids were prescribed more, with morphine and fentanyl being utilized by 19.0% and 7.10%, respectively. 

**Figure 4:**
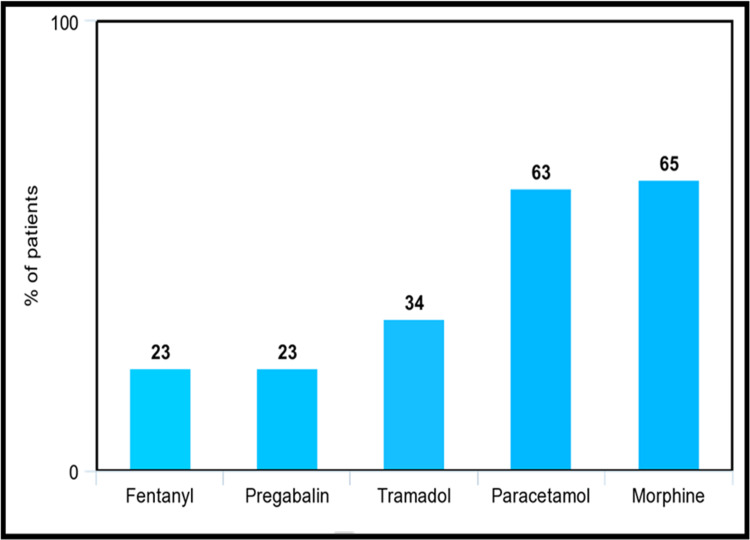
Most common analgesics prescribed for severe pain as a percentage of patients The specific analgesic drugs that were utilized by patients experiencing severe pain, represented by the percentage of patients utilizing them as shown in this bar chart.

As for severe pain, in Figure 4, morphine became the most prescribed drug, overtaking paracetamol. Morphine was prescribed for 65.0% of patients and paracetamol for 63.0%. Tramadol was used by 34.0% of the sample. Finally, fentanyl and pregabalin, the strongest two opioids as per the WHO classification, increased in prescription for severe pain compared to mild and moderate pain, with both being prescribed to 23.0% of patients.

The oral route of administration was the most prescribed, with 128 (64.0%) patients utilizing it. This was followed by the intravenous IV route utilized by 92 (46.0%) patients. Lastly, the transdermal route was utilized by 27 (13.5%) patients, as only fentanyl is available as a transdermal formulation (Table 3).

**Table 3:** Route of administration of analgesics by the percentage of patients The three routes of administration used for utilizing analgesics, represented as the number and percentage of the sample utilizing it. IV: intravenous

Route	Number and percentage (%)
Oral	128 (64.0%)
IV	92 (46.0%)
Transdermal	27 (13.5%)

## Discussion

This is the first study to determine the prescription patterns of analgesic drugs in pain management among palliative care patients at a hospital (SQUH) in Oman. Regarding the socio-demographic profile, specifically gender, female patients (59.5%) were more prevalent than males (40.5%) in the current study. A study that explored the preference for palliative care in cancer patients found that females were three times more likely to consider palliative care than males [15]. This same study suggested that societal norms played a role in this divide, where males consider palliative care a weakness and a sign of giving up [15]. A study exploring patients' attitudes toward palliative care and the barriers that prevent them from seeking medical help needs to be conducted in Oman to determine if there is a gender difference regarding the perception of palliative care.

Pain is the most common symptom experienced by palliative care patients [5]. A study by Wilson et al. found that 268 (70.3%) palliative care patients experienced pain, regardless of severity [16]. Among those 268 patients, 129 (48.1%) experienced severe pain. In our study, exactly 50.0% of the sample experienced severe pain; thus, our findings were similar. In our study, patients experienced more mild pain (29.0%) than moderate pain (21.0%). This differs from the study by Menezes et al., in which a higher number of patients experienced moderate pain (38.8%) than mild pain (12.5%) [17]. Those findings could be due to the differences in the stages and, thus, the severity of cancer. Cancer is a disease with multiple stages; thus, our sample may have had a higher number of patients in the earlier stages, hence a higher number of patients experiencing mild pain than moderate pain.

In the present study, patients with hematological and oncological malignancies were considered. Patients with oncological malignancies were much higher and represented most of the sample at 95.5%. This could be because cancer is the most common disease among palliative care patients [3]. Another justification for this stark difference, which needs addressing by healthcare professionals, is the difficulty of predicting disease progression. A study stated that predicting the progression of hematological malignancies is, by nature, more difficult than predicting cancer progression, as prognosis among hematologic diseases can differ substantially within the same diagnosis [18]. Thus, fewer patients with hematological malignancies are classified as needing palliative care. Therefore, international and nationwide efforts are needed to address this issue.

In terms of cancer, breast cancer was the most common (36.5%), followed by colorectal cancer (13.0%). Breast cancer is the most common in Oman, followed by non-Hodgkin lymphoma and colon cancer [1]. Therefore, our sample has similarities to the nationwide statistics, though we did not report as many incidences of non-Hodgkin lymphomas.

Analgesic drugs are the most prescribed drugs for palliative care patients [13]. As per both the WHO and modified WHO pain ladder, the order of analgesic classes in terms of increasing pain relief is as follows: non-opioids (paracetamol and NSAIDs), weak opioids (codeine and tramadol), strong opioids (morphine and fentanyl), and anticonvulsants/neuropathic pain agents (pregabalin and gabapentin) [10-11]. Table 2 demonstrates the different classes and types of analgesics prescribed to our sample. As seen, paracetamol was the most prescribed analgesic in general and was utilized by 128 patients (64.0%). This was similar to the study by Menezes et al., which also found paracetamol to be the most prescribed drug and was utilized by 62.2% of the patients. The study by Praveen et al., on the other hand, differed from those two. In that, it found morphine to be prescribed to 58.0% of their sample and paracetamol to 44.5% of their sample. Since paracetamol is effective for mild pain, it is expected to be utilized by a sizeable number of patients in our sample. In addition, paracetamol can also be used as an adjuvant to strong opioids for moderate to severe pain, and thus it is widely used for palliative care patients. Furthermore, paracetamol can be administered through many routes, such as oral, rectal, intravenous (IV), and other routes. Hence, this also supports why it is the most prescribed analgesic drug in the sample. The second most prescribed analgesic drug in the sample was morphine, which 73 (36.5%) patients used. Tramadol came in third and was utilized by 61 (30.5%) patients. The studies conducted by Menezes et al. and Praveen et al. had some similarities and differences with our results. Menezes et al. identified tramadol as the second-most prescribed analgesic (43.2%), followed by morphine (39.8%) [17]. Praveen et al. had morphine as the most prescribed analgesic (58.0%), followed by paracetamol, and then tramadol in the third (39.0%) [19]. While those differences are noticeable, they can all be explained through the WHO analgesic ladder and the nature of the samples in the studies. As per the ladder, tramadol is used mostly for moderate pain as it is a weak opioid. Morphine, on the other hand, is used for severe pain. Therefore, given that 50.0% of our study sample experienced a severe degree of pain, it can be expected that morphine would be heavily prescribed. In addition, more of our sample experienced mild pain rather than moderate pain; hence, it can also be understood why tramadol is less prescribed than morphine, given that it is mostly used for moderate pain. In terms of classes of analgesic drugs, apart from paracetamol, opioids were the most prescribed, followed by anticonvulsants and, finally, NSAIDs.

Multiple routes of administration are available for analgesic drugs. In our study, three routes of administration were utilized: the oral route (64.0%), the IV route (46.0%), and the transdermal route (13.5%). The literature supports those findings, which suggest that the oral route is the most common and preferred method due to its cost-effectiveness, convenience, and lack of invasiveness [20]. In addition, the most prescribed analgesics in this study, such as paracetamol and morphine, can be given orally, thus justifying why it is the most common route. The IV route is the second-most utilized route, despite its invasiveness. Drugs, including paracetamol, tramadol, and morphine, can be given through the IV route. The transdermal route was the least utilized in our study, and this is because only fentanyl, among the drugs in our study, can be given through this route as a patch.

Though this study was only intended to focus on a single center (SQUH), the findings cannot be generalized to all the hospitals in Oman. In addition, only analgesic drugs from the most recent admission were considered; thus, there may be differences with previous admissions. One other limitation is the idea of prescription vs. utilization. What is prescribed is not necessarily utilized, as some patients may not be compliant; therefore, this may cause some errors (though stronger analgesics must be given at admission). Lastly, the retrospective nature of this study may result in some missing past data and the loss of some patients on follow-up.

It is recommended that similar retrospective studies be conducted in other hospitals in the country and the region. In addition, a prospective study on this topic may be conducted using the Edmonton Symptom Assessment System tool [21]. Similar studies can also be conducted on other life-threatening diseases that require palliative care.

## Conclusions

Analgesics are the most commonly prescribed medications among PC patients worldwide. Different hospitals in the region have different prescribing patterns, and so this study explored the prescription patterns of analgesic drugs for PC patients at SQUH specifically.

From the data collected through the SQUH TrakCare system, it was found that cancer, specifically breast cancer, was the most common disease among the sample. Paracetamol was the most prescribed analgesic drug overall, whereas morphine was the most prescribed drug for severe pain. The oral route of administration was the most utilized. This study's findings were similar to those of other hospitals in the region and worldwide, though there were some minor differences. In addition, the drugs prescribed for the various pain levels are in accordance with the WHO guidelines, as observed through the comparison with the WHO analgesics ladder, demonstrating the significance of this study's findings. Since many other life-threatening diseases could require PC, future studies could focus on the prescription patterns of analgesics for those diseases, beyond this study's scope, which focused purely on cancer and hematological diseases. Furthermore, future studies can evaluate the prescribing patterns among other tertiary hospitals in Oman, evaluating their similarity to SQUH and the WHO guidelines.
